# bletl ‐ A Python package for integrating BioLector microcultivation devices in the Design‐Build‐Test‐Learn cycle

**DOI:** 10.1002/elsc.202100108

**Published:** 2022-03-01

**Authors:** Michael Osthege, Niklas Tenhaef, Rebecca Zyla, Carolin Müller, Johannes Hemmerich, Wolfgang Wiechert, Stephan Noack, Marco Oldiges

**Affiliations:** ^1^ Forschungszentrum Jülich GmbH Jülich Germany; ^2^ Institute of Biotechnology RWTH Aachen University Aachen Germany; ^3^ Computational Systems Biotechnology (AVT.CSB) RWTH Aachen University Aachen Germany

**Keywords:** BioLector, feature extraction, growth rate, microbial phenotyping, uncertainty quantification

## Abstract

Microbioreactor (MBR) devices have emerged as powerful cultivation tools for tasks of microbial phenotyping and bioprocess characterization and provide a wealth of online process data in a highly parallelized manner. Such datasets are difficult to interpret in short time by manual workflows. In this study, we present the Python package bletl and show how it enables robust data analyses and the application of machine learning techniques without tedious data parsing and preprocessing. bletl reads raw result files from BioLector I, II and Pro devices to make all the contained information available to Python‐based data analysis workflows. Together with standard tooling from the Python scientific computing ecosystem, interactive visualizations and spline‐based derivative calculations can be performed. Additionally, we present a new method for unbiased quantification of time‐variable specific growth rate ${\vec{\mu }}_{t}$ based on unsupervised switchpoint detection with Student‐t distributed random walks. With an adequate calibration model, this method enables practitioners to quantify time‐variable growth rate with Bayesian uncertainty quantification and automatically detect switch‐points that indicate relevant metabolic changes. Finally, we show how time series feature extraction enables the application of machine learning methods to MBR data, resulting in unsupervised phenotype characterization. As an example, Neighbor Embedding (t‐SNE) is performed to visualize datasets comprising a variety of growth/DO/pH phenotypes.

**Abbreviation**
bletl: BioLector Extract, Transform, Load
BS: backscatter
DBTL: Design ‐ Build ‐ Test ‐ Learn
DO: Dissolved Oxygen
FAIR: Findability, Accessibility, Interoperability, Reusability
IPTG: Isopropyl‐β‐D‐thiogalactopyranosid
MAP: maximum *a‐posteriori*
MBR: microbioreactor
MCMC: Markov‐chain Monte Carlo
NUTS: No‐U‐Turn Sampler
PCA: Principal Component Analysis
t‐SNE: t‐distributed Stochastic Neighbor Embedding

## 1 INTRODUCTION

The development of innovative bioprocesses is nowadays often carried out in a Design ‐ Build ‐ Test ‐ Learn 31 (DBTL) cycle [1], where fast iterations of this cycle are desired to shorten development times and therefore save costs. This acceleration can be enabled by modern genetic engineering tools, lab automation and standardized data analysis pipelines. One aspect in the “Test” part of the DBTL cycle of a bioprocess is the cultivation of the microorganisms to be tested. This is often performed in microbioreactor systems, since they provide a good balance between adequate throughput and scalability of the results to laboratory scale bioreactors as the gold standard [2, 3].

A typical microbioreactor system will provide transient monitoring of biomass formation, dissolved oxygen, pH, and fluorescence. Usually, the researcher has access to additional environmental data such as temperature, shaking or stirrer frequencies, humidity, and gas atmosphere. Analyzing this heterogeneous, multi‐dimensional data in a quick and thorough manner can be challenging, especially since vendor software often covers only a limited amount of use cases.

From our experience, most researchers try to alleviate such problems by employing spreadsheets of varying complexity, available with various software solutions. While presenting an easy way for simple calculations and visualizations, extensive analysis of the data quickly results in hardly maintainable documents, which are challenging for colleagues to comprehend, error‐prone and easy to break. Most importantly, such multi‐step manual data transformations do not comply with the FAIR data principles, because it is often not accessibly documented which operations were applied. In contrast, our bletl package directly addresses the accessibility aspect and creates incentives to, for example, retain the original data.

**PRACTICAL APPLICATION**

The bletl package can be used to analyze microbioreactor datasets in both data analysis and autonomous experimentation workflows. Using the example of BioLector datasets, we show that loading such datasets into commonly used data structures with one line of Python code is a significant improvement over spreadsheet or hand‐crafted scripting approaches. On top of established standard data structures, practitioners may continue with their favorite data analysis routines, or make use of the additional analysis functions that we specifically tailored to the analysis of microbioreactor time series.

Particularly our function to fit cross‐validated smoothing splines can be used for *on‐line* signals from any microbioreactor system and has the potential to improve robustness and objectivity of many data analyses. Likewise, our random walk based ${\vec{\mu }}_{t}$ method for inferring growth rates under uncertainty, but also the time‐series feature extraction may be applied to *on‐line* data from other cultivation systems as well.

Our package can be installed from PyPI, its code is available on https://github.com/JuBiotech/bletl and extensive documentation online at https://bletl.readthedocs.io.

Automated data analysis pipelines solve this problem by removing the repetitive and error‐prone manual workflows in favor of standardized workflows defined in code. Such workflows offer many advantages, if done correctly: (a) data processing is clearly understandable and documented; (b) every step is carried out for every input data file in the same way, guaranteeing the integrity and reproducibility of the results; (c) data processing can be autonomously started after data generation; and (d) such a pipeline can be run on remote systems, which is especially useful for computational demanding calculations. Such data analysis pipelines are routinely used, for example, for sequencing data [4], but seldom used for microbioreactor data. While multiple Python packages for working with microplate reader data can be found on PyPI [5, 6], we would like to emphasize that microplate readers are typically not designed for cultivation and *on‐line* measurement. Furthermore, most of these implementations are tailored to specific experimental designs or research applications, making them less useful for application in other fields.

Automated data analysis opens up possibilities for at‐line analysis and subsequent intervention during an experiment: Cruz Bournazou et al. report a framework for online experimental redesign of 10 mL 2mag microbioreactors using a data pipeline implemented in MATLAB [7]. Prior work employing *at‐line* data processing from the BioLector platform includes Jansen et al. [8] where a Python‐based process control system was employed to control pH and enzyme addition with a liquid handling robot, but the code was not released alongside the publication. Hemmerich et al. [9] published MATLAB scripts for growth rate calculation from BioLector data, but the code acts on the XLSX file format that cannot be obtained without a human‐in‐the‐loop. A search for “BioLector” on PyPI, the most popular package registry in the Python ecosystem, revealed two Python packages BioLectorPy for plotting, and getgrowth for growth rate calculation from BioLector data, but the corresponding code is not in a public repository. These examples show that while automatable parsing and processing of BioLector data have been reported in literature, all prior work is tailored to specific use cases and neither unit‐tested nor universally applicable.

In this study, we introduce bletl as a first‐of‐its‐kind open‐source Python package for reliable standalone and *at‐line* parsing and analysis of BioLector microbioreactor data. The name bletl is inspired by the fact that it simplifies the implementation of extract, transform, load data processing workflows specifically for BioLector datasets. It is capable of parsing raw BioLector data without involving vendor software, making necessary calibrations for fluorescent‐based measurement of pH and dissolved oxygen, and presenting all measurement, environmental and meta data in the easily accessible, DataFrame format from the popular pandas library [10, 11]. Currently, bletl is designed to parse data from the devices BioLector I, II, and Pro manufactured by Beckman Coulter Life Sciences, but its general design and methods can be applied also for other devices. Specifically, its analysis submodules are built on top of standard data structures from the Python ecosystem, thereby providing the user with generally applicable data analysis routines for high‐resolution biological time series data. For example, we provide functions for data smoothing by cross‐validated spline approximation, growth rate analysis and time series feature extraction. Building on top of our recent work on uncertainty quantification and calibration modeling [12], we introduce a new method for Bayesian analysis of time‐variable specific growth rate and benchmark the objectivity of the method on a synthetic dataset in comparison with commonly used alternative growth rate calculation methods. In addition to an extensive documentation and automated software tests, we provide application examples on a dataset without biological surprises, such that it is well suited for method understanding. With bletl , scientists using microbioreactors have a powerful tool to make their data analysis less cumbersome and error‐prone, while they can directly benefit from state‐of‐the‐art machine learning techniques.

## 2 MATERIALS AND METHODS

### 2.1 Core package

The bletl package includes the data structures that are common to datasets originating from BioLector I, II and Pro microbioreactors. Parsing of raw data is deferred to *parsers* that may implement logic that is specific to a certain BioLector model or file type. Core functionality and analysis methods are extensively tested by automated testing pipelines for Python versions 3.7, 3.8, and 3.9. Support for Python 3.10 will be added as soon as all dependencies are compatible.

#### 2.1.1 Parsing and data structures

**FIGURE 1:**
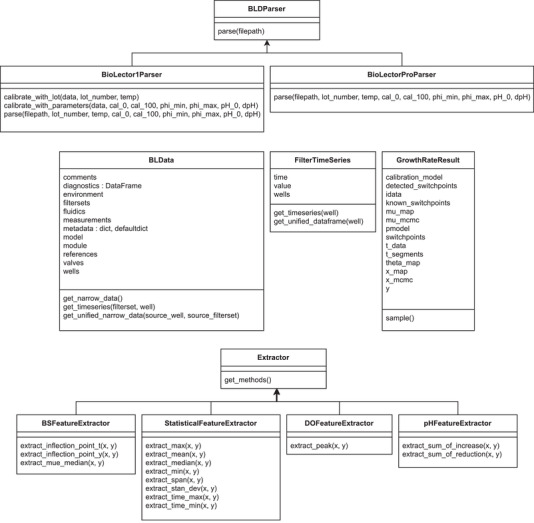
UML diagram of relevant classes in the bletl package. Device‐specific parsers are implemented by inheriting from a common base class that defines the interface needed for integration with the bletl.parse function (top). Data structures for an entire BioLector dataset (BLData) and data from one filterset (FilterTimeSeries) are used to group relevant attributes and provide methods for convenient data access and summarization. Results of the ${\vec{\mu }}_{t}$ method are managed as GrowthRateResult objects, providing various attributes needed for visualization. Custom feature extraction methods, for example to extract model‐based parameters like in [13], may be implemented by inheriting from the Extractor base class

Parsing of raw data typically begins with a call to the bletl.parse function, which first determines the file type from its content. The parsing procedure does not only ingest the data into accessible Python data structures, but also takes care of re‐naming tabular data columns to a standardized naming scheme and type‐casting values to integer, float, or string types. After the BioLector model and file type version are identified, a matching *parser* is selected, thereby enabling a plug‐in system for specialized parsers for different file type versions or the new BioLector XT device. The parsing logic is highly dependent on the BioLector model, but generally follows the pattern of first separating the file header of metadata from the table of measurement data. Logical blocks of information, such as the table of filtersets are then parsed into pandas.DataFrame objects. These tabular data structures are collected as attributes on a bletl.BLData object which is returned to the user. The BLData class is a Python dictionary data type with additional properties and methods. Via its properties, the user may access various DataFrame tables of relevant metadata, including the aforementioned tables of filtersets, comments or environment parameters such as chamber temperature or humidity. Users of prototype BioLectors, or the new BioLector XT are invited to contribute parsers to https://github.com/JuBiotech/bletl. A UML diagram of the relevant classes is shown in Figure 1.

Key‐value pairs in the BLData dictionary are the names of filtersets and corresponding FilterTimeSeries objects. This second important type from the bletl package hosts all measurements that were obtained with the same filterset. Like the BLData class it provides additional methods such as FilterTimeSeries.get_timeseries for easy access of time/value vectors.

### 2.2 Analysis methods

In submodules of the bletl package, various functions are provided to facilitate higher‐throughput and automated data analysis from bioprocess timeseries data.

One often used feature is the find_do_peak function that implements a Dissolved Oxygen (DO)‐peak detection heuristic similar to the one found in the m2p‐labs *RoboLector* software. The DO‐peak detection algorithm finds a cycle number corresponding to a DO rise, constrained by user‐provided threshold and delay parameters.

Additional, more elaborate analysis functions were implemented to allow for advanced data analysis or experimental control.

#### 2.2.1 Spline approximations

To accommodate for the measurement noise in *on‐line* measured time series, various smoothing procedures may be applied to the raw signal. A popular choice for interpolation are spline functions, specifically *smoothing splines* that can reproduce reasonable interpolations without strong assumptions about the underlying relationship. With bletl.get_crossvalidated_smoothing_spline we implemented a convenience function for fitting smoothing splines using either scipy or csaps [14, 15] for the underlying implementation Code 1. Both smoothing spline implementations require a hyperparameter that influences the amount of smoothing. Because the choice of the smoothing hyperparameter strongly influences the final result we automatically apply stratified k‐fold cross‐validation for determining its optimal value. The implementation can be found in the code repository of the bletl project [16].

**Code 1: Code to obtain a cross validated smoothing spline for pH data**

The user must provide vectors for time and value of a time series to which the spline will be fitted (line 1). A spline is then obtained in line 2, while specifying the preferred spline method. The returned object behaves like a SciPy spline and can, for example, be called on a vector of high‐resolution time points to evaluate interpolated values.


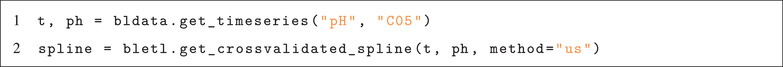


#### 2.2.2 Growth rate analysis

A “calibration‐free” approach to calculate time‐variable specific growth rate μ(t) (1) relies on the previously introduced spline approximations, combined with the popular assumption of a linear backscatter $Y_{BS}$ versus biomass X relationship (2).

(1) $$\mu \left(t\right)=\frac{dX}{dt}\cdot \frac{1}{X(t)}=\frac{\dot{X}\left(t\right)}{X(t)}$$

(2) $$\begin{array}{c} \begin{array}{cc} Y_{BS}\left(t\right) & =a\cdot X(t)+b\\ \Leftrightarrow X(t) & =\frac{Y_{BS}\left(t\right)-b}{a} \end{array} \end{array}$$

Substituting the biomass X(t) in (1), the slope parameter a cancels out such that only a “blank” b and the measured backscatter $Y_{BS}\left(t\right)$ are needed for a specific growth rate calculation (3).

(3) $$\begin{array}{c} \begin{array}{cc} \Leftrightarrow \mu (t) & =\frac{1}{a}\cdot \frac{d(Y_{BS}\left(t\right)-b)}{dt}\cdot \frac{a}{Y_{BS}\left(t\right)-b}\\ \Leftrightarrow \mu (t) & =\frac{1}{Y_{BS}\left(t\right)-b}\cdot \frac{d(Y_{BS}\left(t\right)-b)}{dt} \end{array} \end{array}$$

Finally, the backscatter curve $Y_{BS}\left(t\right)$ can be approximated by a smoothing spline $S_{\mathrm{YS}},\mathrm{blanked}\left(t\right)$ to obtain a differentiable function (4).

(4) $$\;\mu \left(t\right)=\frac{{\dot{S}}_{\mathrm{YS}},\mathrm{blanked}\left(t\right)}{S_{\mathrm{YS}},\mathrm{blanked}\left(t\right)}$$

An alternative approach is to construct a *generative* model of the biomass growth. In essence, the time series of observations is modeled as a deterministic function of an initial biomass concentration $X_{0}$ and a vector of specific growth rates ${\vec{\mu }}_{t}$ at all time points where observations were made. The structure of this model assumes exponential growth between the time steps, which is a robust assumption for high‐frequency time series such as the ones obtained from BioLector processes.

(5)

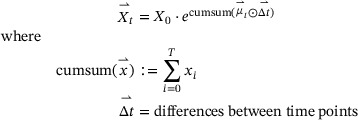




The convenience function bletl.growth.fit_mu_t creates the generative ${\vec{\mu }}_{t}$ model (5) from user‐provided vectors of observation time points $\vec{t}$, backscatter values $\vec{y}$ and a calibration model built with the calibr8 package. The calibration model used for this study is based on an asymmetric logistic function of the log‐biomass concentration. Most importantly it describes the distribution of backscatter observations that can be expected from an underlying biomass concentration, thereby enabling uncertainty quantification that accounts for precision of the measurement method. A thorough introduction can be found in [12], but generally a calibration model can be constructed in three steps:

1. **Acquisition of calibration data**, preferably singular replicates at a high number of concentrations ranging three orders of magnitude up to 2−3× the biomass concentration expected in experiments.
2. Definition of the model class and **parameter estimation**.
3. **Quality checks** and iteration over 1‐3 until the quality of the data and model is satisfactory.

For calibrations of biomass/backscatter relationships in the BioLector system we recommend the procedure described in the methods section of [12].

To contrast this approach from the smooth, continuous μ(t) from (4) we use the ${\vec{\mu }}_{t}$ notation to underline that the approach discretizes the growth rate into a step function. The model is built with the probabilistic programming language PyMC [17, 18] and an optimal parameter set, the maximum *a‐posteriori* (MAP) estimate, is found automatically by optimization. Additionally, the user may decide to perform Markov‐chain Monte Carlo (MCMC) sampling using advanced sampling algorithms such as No‐U‐Turn Sampler (NUTS) from the PyMC [17] package to infer probability distributions for the model parameters $X_{0}$ and ${\vec{\mu }}_{t}$. Both MAP and MCMC parameter estimation methods are based on the log‐posterior probability of the model. For MAP estimation the PyMC framework obtains gradients by auto‐differentiation and employs the L‐BFGS‐B minimization algorithm from SciPy. Whereas MAP estimation yields one point in the parameter space with relatively little computational effort, MCMC parameter estimation is more expensive, but yields thousands of points (samples) that approximate the joint posterior probability distribution. For the application shown here, the most relevant advantages of MCMC parameter estimation are the uncertainty quantification of model parameters and the more reliable convergence compared to optimization. For a more comprehensive introduction to Bayesian methods we recommend [19].

**FIGURE 2:**
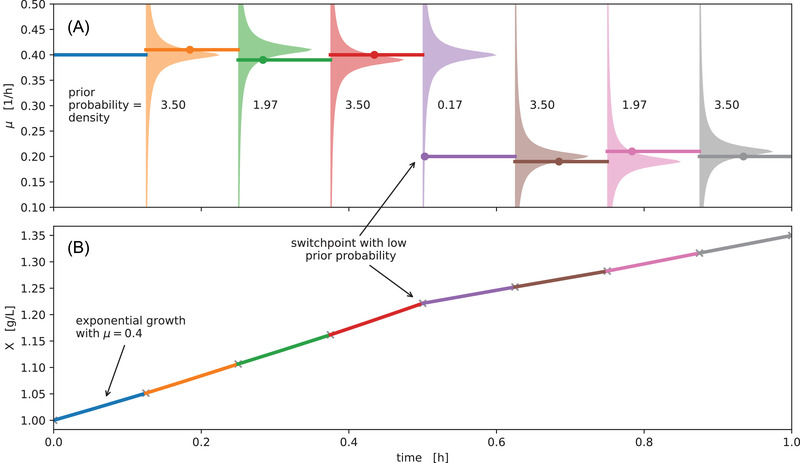
Switchpoint detection with a Student‐t random walk. A microbial growth curve with fluctuating specific growth rate μ may be discretized into segments of exponential growth with constant growth rate (solid lines). Modeling such a sequence of growth rates with a random walk assigns prior probabilities to every value of $\mu \in \vec{\mu }$, centered on the values of previous iterations (A, colored areas). When fitting the model these prior probabilities “pull” subsequent values in $\vec{\mu }$ towards each other, leading to a smoothing and counteracting overfitting. Using a fat‐tailed Student‐t distribution for the random walk prior, the penalty for large jumps is less extreme compared to a Normal distribution, thereby allowing for jumps in the random walk (5th segment)

In the generative $\mu_{t}$ model, the vector of growth rates is modeled with either a Gaussian or Student‐t distributed random walk (Figure 2). This does not only result in a smoothing of the growth rate vector, but enables additional flexibility with respect to switchpoints in the growth rate. A drift_scale parameter must be given to configure the random walk with a realistic assumption of how much growth rate varies over time. Small drift_scale corresponds to the assumption that growth rate is rather stable, whereas large drift_scale allows the model to describe a more fluctuating growth rate distribution. On the technical level, the drift_scale parametrizes the width of the Student‐t random walk prior (6), pulling the values of the random walk closer together, since the prior‐probability is a term in the log‐posterior probability of the model.

(6) $$\begin{array}{ccc} \log(p_{\mathrm{prior}}({\vec{\mu }}_{t})) & = & \sum_{i=0}^{T-1}{\mathrm{logpdf}}_{\mathrm{StudentT}}\\ & & \cdot \;({\vec{\mu }}_{t,i+1}|{\vec{\mu }}_{t,i},\mathrm{drift}\_\mathrm{scale},\nu ) \end{array}$$

Additionally, the user may provide previously known time points at which growth rate switches are expected. Examples of such switchpoints are the time of induction, occurrence of oxygen limitation or the time at which the carbon source is depleted. If ${\vec{\mu }}_{t}$ is described by a Student‐t random walk, switchpoints can be detected automatically by inspecting the prior probabilities of the estimated growth rate in every segment (Figure 2). Our implementation automatically classifies elements of $\vec{\mu }$ as switchpoints as soon as their prior probability is <1%.

While there has been prior work on using random walks and cumulative sums for outlier detection [20, 21, 22], we are not aware of prior work using Student‐t random walks for the unsupervised detection of changepoints in time series data. The general idea of modeling exponential growth from a random‐walk of the growth rate was inspired by early versions of the “Rt.live” model of COVID‐19 effective reproduction numbers [23].

#### 2.2.3 Feature extraction

The bletl.features submodule implements functions for the automated extraction of both biologically and statistically motivated time series features. An abstract Extractor class may be inherited to implement feature extraction of characteristic features such as DO peaks. Additionally, our TSFreshExtractor uses the open source Python package tsfresh [24] to extract hundreds of features from variable length time series automatically. Examples of such features are times of min/max values, quantiles, autocorrelation lags or fourier transform coefficients.

Starting from a bletl.BLData object containing one or more FilterTimeSeries, the bletl.features.from_bldata function extracts features from multiple filtersets using a user‐specified mapping of Extractor objects. Optionally, a dictionary of well‐wise cycle numbers can be passed to truncate time series to the relevant cycles. The results are returned as a DataFrame for maximal compatibility with downstream analysis operations. For details on the implementation we refer to the code and documentation [16, 25].

Code 2 shows how the function is applied to our demonstration data set. The resulting DataFrame comprised 2343 feature columns for each of the 48 wells in the input data. Feature columns with NaN, ±∞ entries or without variability in their values were dropped, resulting in 1282 features available for further analysis.

**Code 2: Code to run feature extraction from three filtersets**

The name of each filterset is mapped to a list of Extractors that may include user‐defined feature extraction implementations. The last_cycle keyword argument can be used to pass a mapping of well IDs to the last relevant cycle numbers. Extracted features are returned in the form of a pandas.DataFrame


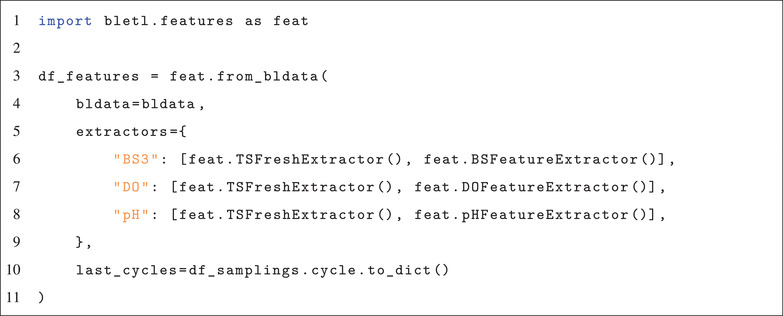


#### 2.2.4 Visualization by t‐SNE

Starting from features extracted with bletl.features we applied the t‐SNE technique to find a two‐dimensional embedding for the visualization of local structure in the dataset [26]. The general idea behind t‐SNE is to find a low‐dimensional arrangement of records from a high‐dimensional dataset, such that the dissimilarity (Kullback‐Leibler divergence) between the two distributions is minimized [26]. The method is frequently used for visualization of local structure in complex datasets such as huge collections of images, single‐cell transcriptomics records, or high‐dimensional latent representations of word embeddings obtained from neural networks [26, 27, 28]. Examples of t‐SNE visualizations are shown in Section 3.4.

For the application of t‐SNE to BioLector time series, we cleaned extracted features such that features with NaN values, or without diversity were removed. After feature‐cleaning, the t‐SNE implementation from scikit‐learn was applied with a perplexity setting of 10 and initialization by PCA. The corresponding code can be found in the Supporting material on GitHub [29].

### 2.3 Media and cultivation conditions

The dataset presented as an application example in this study was obtained in an automated cultivation workflow on the previously described microbial phenotyping platform. 48 cultures of *Corynebacterium glutamicum* ATCC 13032 harboring the pPBEx2[30]‐based plasmid pCMEx8‐NprE‐Cutinase (GenBank accession number OL456171) were cultivated in CGXII medium (recipe as in [12]) with different carbon sources. Carbon sources were prepared as C‐equimolar, random combinations of $8\;\frac{g_{C}}{L}$ glucose, fructose, maltose, sucrose, gluconate, lactate, glutamate or *myo*‐inositol. For every well except A01, where glucose was the sole carbon source, three different carbon sources were chosen at random. A total of 140 µL of C‐equimolar carbon source stock were added to each well. The 140 µL were split into seven parts of 20  µL and such that at least one part was used for each selected carbon source and the remaining four parts were assigned randomly. The resulting media composition in terms of pipetted volume, and carbon mass per microliter can be found in Section 4.1.

Cultivation was done as previously described [31]. Briefly, 800 µL CGXII medium were inoculated to an optical density at 600 nm of 0.2. Cultures were grown in a MTP‐48‐BOH 1 FlowerPlate in a BioLector Pro (both Beckman Coulter Life Sciences, USA) at 1400 rpm, 30 °C and ≥85% humidity. Expression was induced autonomously with 10 µL isopropyl‐β‐D‐thiogalactopyranosid (IPTG) (final concentration 100 µM) when cultures reached a backscatter value of 5.82, corresponding to approximately $4\;\frac{g_{CDW}}{L}$. Culture from each well was harvested 4 h after induction.

## 3 RESULTS AND DISCUSSION

### 3.1 Basic visualization workflow

Every analysis begins with loading data into a structure that can be used for further analysis. In the case of a BioLector experiment, the data are multiple tables that hold information about filtersets, environment variables such as temperature or humidity, as well as the well‐wise measurements. In most cases the result files already contain relevant meta information such as lot number or process temperature and parsing them with bletl comes down to a single line of Python (Code 3).

**Code 3**: **Parsing of a BioLector result file**

The bletl.parse function automatically determines the file type (BioLector I, II or Pro) and applies calibration of optode measurements based on lot number and temperature from the file. Optionally, lot number and temperature, or calibration parameters may be passed to override the values from the file. The function can also process a list of result file paths and automatically concatenate them to a single BLData object.


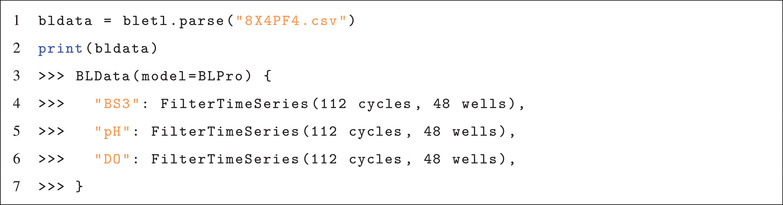


The bletl.BLData type is a dictionary‐like data structure into which results are loaded. It has additional properties through which process metadata and measurements that are not tied to individual wells can be obtained. Its text representation, which appears when the object is displayed in an interactive Jupyter notebook session shows the names of filtersets and the amount of data they contain (Code 3).

Elements in the BLData object are the FilterTimeSeries, that contain the well‐wise measurements. The simplest way to access the time series of a particular filterset and well is via the BLData.get_timeseries(well, filterset) or FilterTimeSeries.get_timeseries(well) methods. Optionally, the user may pass cycle number via the last_cycle keyword‐argument of get_timeseries to retrieve only the data up to that specific cycle number. This is useful in situations where wells were sampled and might only be analyzed up to the sampling time point.

In the dataset presented here, cultures were induced and sampled by a robotic liquid handler. Induction was triggered *at‐line* based on latest backscatter observations and sampling was performed 4 h after the induction events Section 2.3. The metadata of these induction and sampling events were logged into an XLSX file and loaded into a pandas.DataFrame for the analysis Table 1.

**TABLE 1:** Excerpt of sampling event log

well	Timestamp	Time	Cycle	Volume	Supernatant_well
**A01**	2020‐07‐21T08:10:04.721Z	13.076466	61	−950	H01
**A02**	2020‐07‐21T07:05:04.299Z	11.993016	56	−950	G01
**A03**	2020‐07‐21T08:10:04.786Z	13.076485	61	−950	F01
**A04**	2020‐07‐21T08:10:04.841Z	13.076500	61	−950	E01
**A05**	2020‐07‐21T06:26:17.384Z	11.346651	53	−950	D01

The meta information about induction and sampling events is important for the analysis, because backscatter, pH and DO observations made after a well was *sacrifice*‐sampled must be truncated before analysis or visualization.

**FIGURE 3:**
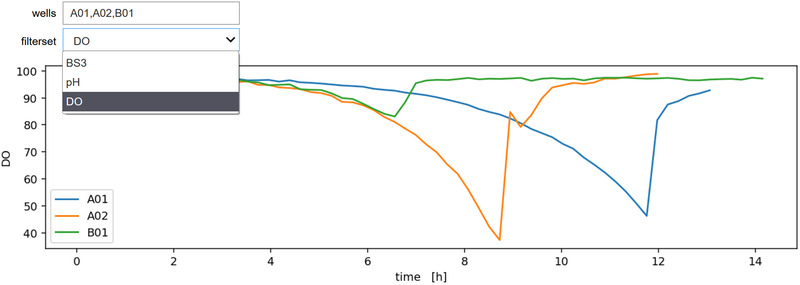
Interactive plot of well‐wise measurements. A plot_custom function, defined in lines 1‐17 takes a comma‐separated text of well IDs and the name of a filterset as parameters for the visualization. In line 4 it iterates over the well IDs to create lines plots of the measurements, passing the number of the last relevant cycle from the event log (Table 1) to truncate the data. Line 21 passes the list of filtersets in the dataset (Code 3) as options for the fs keyword‐argument of the plotting function, thereby populating the dropdown menu

With the data structures provided by our bletl package, the data analysis workflow for a BioLector experiment is no different to any standard data analysis performed with Python. Such analyses are often driven by interactive exploration of the data. This is facilitated by interactive plots using helper functions from, for example, the ipywidgets library. Code 4 and Figure 3 show the code and resulting interactive plot of measurement results from a BioLector dataset. The ipywidgets library is used to wrap a plotting function and create interactive input elements for selecting the filterset and wells to show.

**Code 4: Use of bletl and ipywidgets to generate an interactive plot**

By using the function get_timeseries, measurements of specified wells and filtersets are extracted from the dataset. The parameter last_cycle is used to truncated the vectors according to the sampling time point.


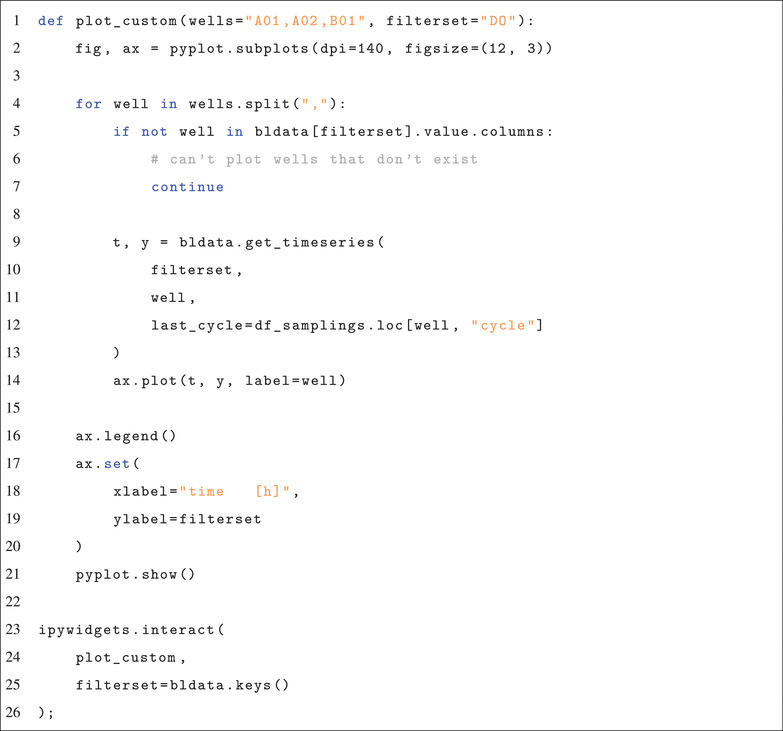


### 3.2 Splines for time series smoothing and derivatives

Optical *on‐line* measurements as those performed by the BioLector are inevitably subject to measurement noise. While the measurement noise of DO and pH signals in the BioLector II/Pro system was greatly reduced compared to the BioLector I model, it still requires special attention in subsequent data analysis procedures. Particularly in automated *at‐line* decision making such as triggered induction or sampling, measurement noise can cause problems with threshold‐based heuristics. With noisy *on‐line* signals, such as optode measurements in BioLector I datasets, smoothing splines can yield more accessible visualizations and allow for finer‐grained comparisons. Furthermore, the slope of the signals may be used for more sophisticated analysis or decisions.

For *at‐line* triggers based on such noisy process values, a smoothing of the signal can increase the reproducibility of detecting, for example, a pH threshold. At the same time, the slope of process values often gives more process insight compared to absolute values alone. For example, a dissolved oxygen tension of 60 % alone is not very meaningful, but the observation of a strong positive slope tells the process engineer that the microbes might grow with reduced oxygen uptake rate. The calculation and visualization of pH and DO slopes is therefore an important tool for process data analysis.

Splines are a popular choice for both smoothing and derivative calculation, because they make few assumptions about the data and are available in most standard data analysis software. There are however multiple flavors of *smoothing splines* and they come with a smoothing parameter whose value has a considerable effect on the results. In bletl.splines we implemented a convenience function that automatically performs k‐fold cross‐validation on the smoothing parameter of either a UnivariateSpline cubic spline from scipy or a UnivariateCubicSmoothingSpline from *csaps* (cf. Section 2.2.1).

**FIGURE 4:**
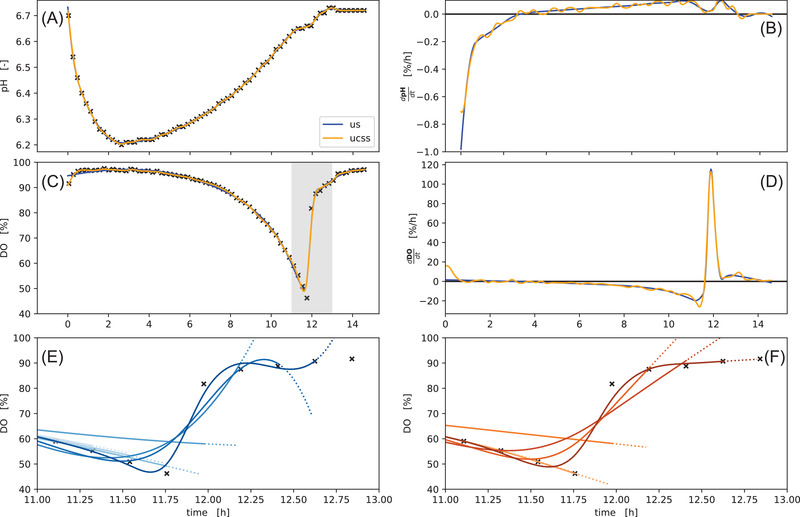
Splines fitted to pH and DO time series. Both spline methods “us” (blue) and “ucss” (orange) were applied to measurements of pH (A) and DO (C). The resulting reconstruction/interpolation is largely identical (A, C) with most notable differences between the methods at the start/end, as well as their derivatives (B, D), where the “ucss” method has considerably more *wiggly* derivatives. With a zoomed‐in time axis (gray area in C) the bottom row **(E, F**) shows extrapolations (dotted lines) of splines fitted to data subsets of different length (solid lines). The “ucss” method extrapolates in straight lines, whereas the “us” method extrapolates with a curvature

In Figure 4 the two spline methods were applied to pH and DO time series of well A01 from our demonstration dataset. Both smoothing spline methods find interpolations (solid lines) of the raw data that are almost indistinguishable. Their 1st derivative however reveals that the UnivariateCubicSmoothingSpline (ucss) from the csaps package is much more wiggly compared to the UnivariateSpline (us) from SciPy.

The bottom row shows a comparison of both methods in a simulated *at‐line* situation where the DO time series grows point by point at the time of the characteristic substrate‐depletion DO‐peak. In this situation the “us” method produces strong alternating positive or negative slopes and curved extrapolation at the end of the curve. In contrast, the splines obtained with the “ucss” method extrapolate with an (almost) constant slope. Taking both scenarios into account, the choice between the “us” and “ucss” depends on the use case. As a rule of thumb, “us” is more suited when steady derivatives are desired, whereas the more stable extrapolation of the “ucss” splines should be preferred for *at‐line* applications.

### 3.3 Growth rate and timeseries analysis

Most cultivations in microbioreactors such as the BioLector are conducted to extract key performance characteristics of the bioprocesses from the *on‐line* measurements. One such performance indicator is the specific growth rate μ. In applications where unlimited exponential growth is observed, a constant maximum specific growth rate $\mu_{max}$ can be calculated by regression with an exponential function [9]. Many processes however do not fulfill this assumption and require a more detailed analysis with time‐variable specific growth rate. Unlimited exponential growth may be terminated by nutrient limitation, or the characteristics of strain and cultivation media may lead to multiple growth phases. For example, overflow metabolism of *E. coli* growth on glucose can lead to an accumulation of acetic acid which is metabolized in a second growth phase. Accordingly, switchpoints in growth rate can indicate limitations, changes in metabolism or regulation.

From temporally highly resolved backscatter observations combined with a detailed biomass/backscatter correlation model, variable specific growth rate can be calculated using our bletl.growth.fit_mu_t function. This model describes the data in a generative fashion by first discretizing time into many segments of exponential growth, followed by simulating the biomass curve resulting from a growth rate that drifts over time. For this it assumes an initial biomass concentration $X_{0}$ and a vector of growth rates $\vec{\mu }$, calculates biomass concentrations deterministically and compares them to the observed backscatter using a calibration model built with the calibr8 package [12]. Parameters $X_{0}$ and $\vec{\mu }$ can be obtained through optimization or MCMC. In this analysis we specified a prior belief in $X_{0}$ centered around 0.25 g/L, corresponding to typical inoculation density for BioLector experiments. The prior for $\vec{\mu }$ is a random walk of either a Normal or Students‐*t* distribution, which pulls the neighboring entries in the growth rate vector closer to each other, resulting in a smooth drift of ${\vec{\mu }}_{t}$ (Section 2.2.2). While this method makes few assumptions about the underlying process and therefore can be applied to many datasets, practitioners wanting to encode process knowledge should also consider differential‐equation based modeling approaches for which Python packages such as pyFOOMB or murefi can be applied [12, 32].

**FIGURE 5:**
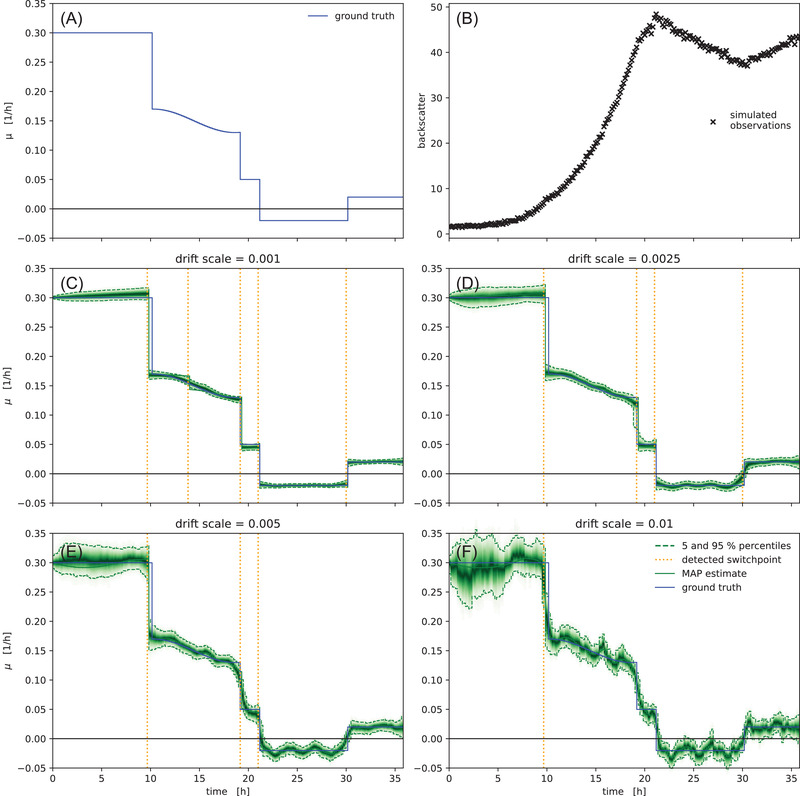
Inference of growth rate from a synthetic dataset. A vector of growth rates (A) exhibiting switchpoints and a smooth fluctuation was used to simulate biomass concentrations (not shown) and corresponding backscatter observations (B). The magnitude of the drift_scale parameter (scale of the Students‐t distribution in the random walk) effects stability, switchpoint detection and uncertainty (C‐F), but in all cases the model fit is unbiased compared to the ground truth. Small drift_scale settings constrain the model to stable growth rates, which are inferred with little uncertainty (C, D). Large drift_scale allows for larger variance in the growth rate, leading to more uncertainty and fewer automatically detected switchpoints (E, F). The green density bands visualize the posterior probability density, with dashed lines marking the 5 and 95% percentiles

To benchmark the objectivity of the method, we generated a synthetic dataset from a vector of growth rates (Figure 5 A, B). The comparison of the inference result with the ground truth (Figure 5) shows that with the correct calibration model it yields unbiased estimates of the underlying growth rate. Figure 5 also shows that the drift_scale parameter can be tuned to reflect an assumption about the stability of growth rate in the model. Low drift_scale constrains the model towards stable exponential growth and correspondingly narrow uncertainties (Figure 5, C, D). Large drift_scale on the other hand encodes the prior belief that growth rate is unstable, leading the model to infer rather unstable growth rates with much higher uncertainty (Figure 5, E, F). In the example from Figure 5 a drift scale of 0.0025 gave the best results and enabled the model to detect all switchpoints without additional false positives.

**FIGURE 6:**
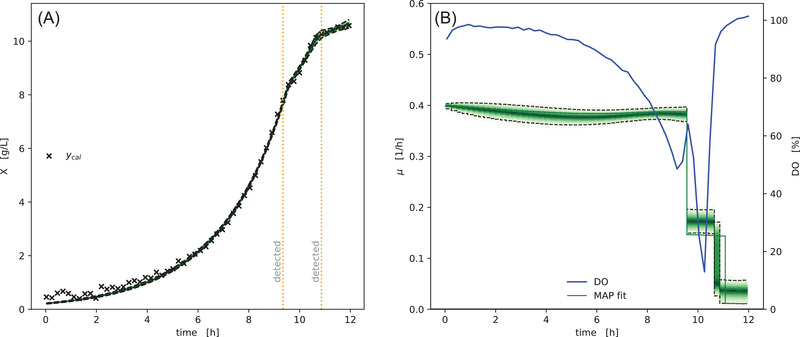
Model prediction of variable growth rate. Biomass concentrations inferred from backscatter observations (A) are well explained by the drift of specific growth rate over time (B). At two timesteps the specific growth rate changed significantly, which resulted in the automatic detection of switchpoints (vertical dashed orange lines). These switchpoints in ${\vec{\mu }}_{t}$ at 9.35 and 10.65 h coincide with changes in the Dissolved Oxygen (DO), indicating a change in cell metabolism. The green density bands visualize the posterior probability density, with dashed lines marking the 5 and 95% percentiles

In Figure 6 we applied our generative ${\vec{\mu }}_{t}$ method to data from well F02 of the example dataset. The carbon source composition in this well were three parts fructose, three parts gluconate and one part lactate, causing a change in growth phase at around 9.35 h. The orange line shows the maximum *a‐posteriori* estimate of ${\vec{\mu }}_{t}$, obtained by optimization. Automatically detected growth rate switchpoints are shown as dashed lines. The green density visualizes the percentiles of the posterior probability distribution of the biomass concentration (left) and growth rate (right). The MAP estimate (orange line),is largely in agreement with the full posterior probability distribution obtained by MCMC. This similarity of MAP and the full posterior distribution is not always the case in Bayesian data analysis, but since the computational runtime to obtain the MAP estimate (seconds) is around 100x lower compared to the runtime of a full MCMC parameter estimation (minutes), it is often the first step when analyzing a new dataset.

The comparison of growth rate over time (right, orange/green) with dissolved oxygen tension (blue) shows that both detected switchpoints in the growth rate fall together with severe changes in the dissolved oxygen concentration. The first switch from $>0.4\;\frac{1}{h}$ to $\approx 0.2\;\frac{1}{h}$ coincides with a temporary increase in DO, whereas the second switch from $\approx 0.2\;\frac{1}{h}$ to $\approx 0.05\;\frac{1}{h}$ falls together with the final rise in oxygen concentration.

One key aspect of growth rate calculation are the assumptions made about the biomass/backscatter relationship. The aforementioned ${\vec{\mu }}_{t}$ method relies on a *calibration model* of backscatter versus biomass concentration to simultaneously describe the relationship and measurement noise with a non‐linear calibration model. This raises the question to what extent growth rate may be quantified with less sophisticated calibrations.

**FIGURE 7:**
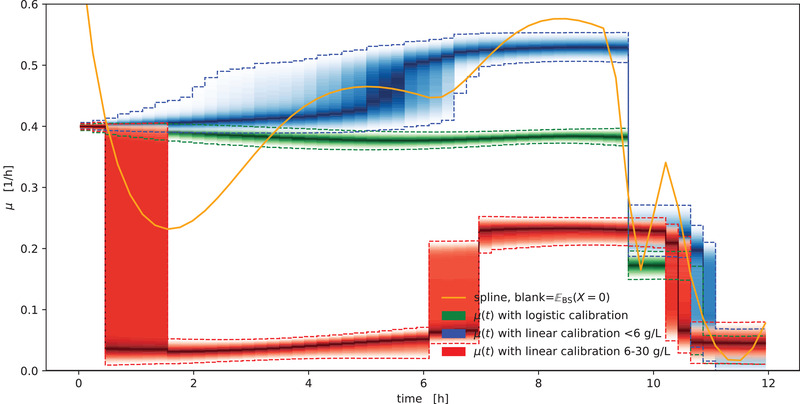
Comparison of growth rate calculation methods. Spline‐based μ(t) growth rate calculation based on blank subtraction (orange) yields a point estimate that fluctuates considerably compared to the “gold standard” of the generative ${\vec{\mu }}_{t}$ method with detailed biomass/backscatter calibration (green). When the generative method is used with linear calibration models, the choice of calibration concentrations and the decision for (blue) or against (red) fixing the intercept at a blank backscatter has considerable effects on the quality of the outcome. The density bands visualize the posterior probability density, with dashed lines marking the 5 and 95 % percentiles

In Figure 7 we compare the results of a “calibration‐free” μ(t) spline approach (Section 2.2.2) with the ${\vec{\mu }}_{t}$ method using linear or logistic calibration models. Note that the “calibration‐free” approach also makes the assumption of a linear relationship between biomass concentration and backscatter observations, just without specifying the slope that cancels out in the growth rate calculation (Section 2.2.2).

Compared to the alternatives, the growth rate curve resulting from the spline method exhibits strong oscillatory artifacts at the beginning of the curve, where the biomass concentration is low. The blue density shows the results of the generative ${\vec{\mu }}_{t}$ method combined with a linear biomass/backscatter calibration that uses calibration data up to 6 g/L and fixes the intercept to a blank value. This model can still detect the switchpoints, but is biased towards considerably higher growth rates (blue). In contrast, a linear calibration with 6–30 g/L that does not fix the intercept parameter to a blank value leads to a strong under‐estimation of the growth rate, largely explained by the lack of fit error of the calibration model (Figure S1). For detailed guidance on the construction and diagnosis of calibration models we refer to [12].

The strength of non‐linearities in the biomass/backscatter relationship may depend on the BioLector model and device at hand, but from Figure 7 we must conclude that a realistic, unbiased biomass/backscatter calibration is indispensable. Such a calibration is not necessarily non‐linear, but when quantitative estimates of specific growth rates are desired, practitioners should first perform a thorough acquisition of calibration data before committing to a possibly biased model. Due to the availability of computational fast, unbiased growth rate quantification with the ${\vec{\mu }}_{t}$ method, we found no convincing advantages of spline‐based growth rate estimation.

### 3.4 Time series feature extraction

It was previously shown that high‐resolution timeseries of culture backscatter can be correlated with product measurements through the use of dimension‐reduction techniques and regression models [13]. With bletl.features we provide an implementation for configurable and automated extraction of large numbers of features from bioprocess timeseries data. These features may be used as the input to a broad spectrum of machine learning pipelines making use of techniques such as dimension reduction, regression, unsupervised visualization or clustering.

**FIGURE 8:**
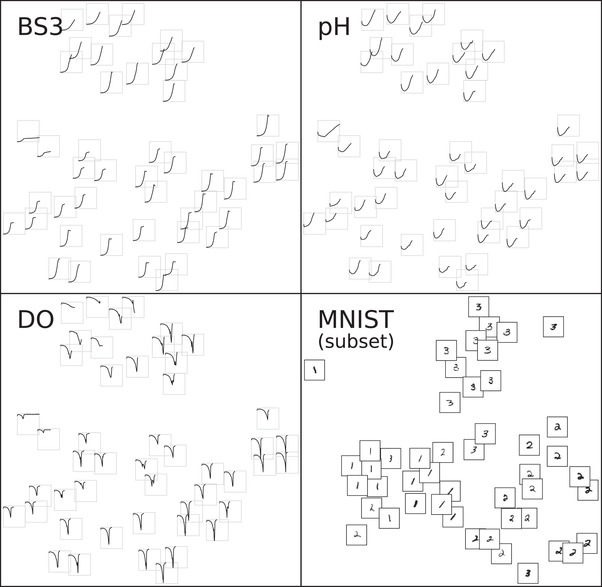
t‐SNE from extracted time series features. Small tiles in subplots BS3, pH and DO correspond to culture wells and were arranged according to the t‐SNE result. The time series inside were truncated at the time of harvest. As with any t‐SNE visualization, the large‐scale arrangement, rotation or axis units are meaningless, since the technique prioritizes local structure. Note that tiles arranged in close proximity are have similar time series characteristics in all three filtersets. For comparison, a t‐SNE embedding of 48 random handwritten 1/2/3 digits from the MNIST dataset is shown in the lower right

To demonstrate how one might use these methods, we applied feature extraction and t‐SNE to the previously introduced dataset to obtain a visualization of local structures in the high‐dimensional data. Probably the most popular example of a t‐SNE visualization are two‐dimensional embeddings of the MNIST handwritten digit dataset. Already with just 48 images from the MNIST dataset, t‐SNE can find a two‐dimensional arrangement of the 784‐dimensional (28 x 28 pixel) records that recovers local similarities between the digits (Figure 8).

However, time series from microbioreactors are typically of unequal length and therefore cannot be fed into the t‐SNE algorithm directly. In a preprocessing step the time series must first be transformed into a fixed number of features. For this demonstration example we extracted initially 2343 features from the full dataset using both biologically motivated, as well as the statistical time series feature extractors. The t‐SNE visualization shown in Figure 8 was then prepared from a cleaned set of 1282 features (Section 2.2.3).

**FIGURE 9:**
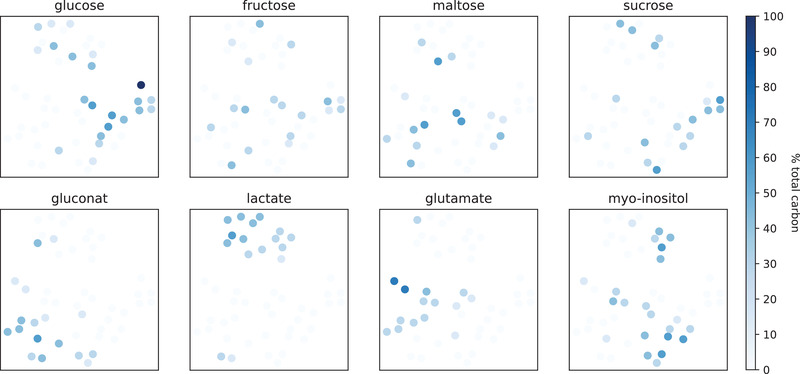
t‐SNE embedding colored by carbon sources. The color intensity encodes the amount of carbon provided via each of the eight carbon sources. Note that with the exception of one well containing 100 % glucose (dark blue, center right) each well contains three carbon sources. Almost all wells that included lactate as a carbon source are closely arranged in the t‐SNE embedding, indicating that they are in close proximity in the high dimensional feature space. Likewise, gluconate or glutamate containing wells are closely arranged. In contrast, none of the monomeric or dimeric sugars lead to characteristic BS/pH/DO phenotypes

Note that the experiment for this application example was purposely designed to not create clusters by, for example, including multiple replicates of the same medium design. Instead, the randomly distributed medium designs were intended to result in growth phenotypes that can be morphed into each other. And indeed there are examples of such morphing in Figure 8, for example the records in the lower‐left are arranged by the strength of the DO‐minimum. Nevertheless, the coloring of the embedding by carbon source composition (Figure 9) reveals that the t‐SNE arrangement is strongly correlated with the presence of gluconate, glutamate and particularly lactate in the cultivation supernatant. Hence, without investigating the metabolic details, the application of unsupervised machine learning methods to this BioLector dataset recovered local similarities and revealed that presence of lactate in the medium lead to a distinctive growth phenotype.

The observation that a t‐SNE of extracted time series features does not only recover similarities between individual wells, but also aspects of the experiment design shows that our feature extraction is a viable solution to make BioLector datasets amenable to machine learning methods. In contrast to the extraction of manually engineered features [13], our feature extraction workflow works out of the box and with few lines of code. The example from Figure 8 shows that t‐SNE can readily deal with large numbers of feature dimensions, even when there are few records in the dataset. Other machine learning methods however may need redundant features to be removed, for which dimension reduction techniques such as linear discriminant analysis could be applied.

## 4 CONCLUDING REMARKS

As we elaborated on in Section 1, bletl is the first (publicly available) Python package to parse and process raw BioLector datasets entirely, without dropping potentially relevant metadata. With the examples in Section 3.1 we showed how bletl thereby makes BioLector datasets accessible to standard Python‐based data analysis workflows. The switch to Python‐based data processing facilitates not only interactive and robust data analysis, but also enables the application of machine learning techniques such as crossvalidated smoothing splines to BioLector datasets. Nevertheless, many scientists who are not yet proficient in Python‐based data analysis workflows might be concerned with the initial complexity of the learning curve. That is one of the reasons why the documentation of the bletl package comes with ready‐to‐use examples. The code of the library is thoroughly tested in automated test pipelines to reduce the chance of unexpected failures.

In Section 3.2 we characterized two strategies for smoothing noisy *on‐line* signals and showed that subtle differences in implementation can have substantial consequences on the results. This again highlights the need for standardized data structures, robust data analysis routines and thoroughly tested, open‐sourced implementations that are distributed through versioned releases. Compared to the state of the art in bioprocess research (see Section 1) where data analysis scripts are seldomly published and rarely versioned, the analysis submodule of our bletl package provides generally applicable, transparent and characterized implementations.

For the analysis of specific growth rate under not necessarily unlimited exponential growth conditions, we presented a random‐walk based ${\vec{\mu }}_{t}$ model that can also detect switchpoints automatically. Within seconds our method determines time‐variable growth rates by optimization and by leveraging state of the art probabilistic machine learning, it also quantifies Bayesian uncertainties. We showed on a synthetic dataset that the method is not only unbiased, but also offers the practitioner a tuning knob for the bias‐variance tradeoff between narrow uncertainties and growth rate flexibility (Figure 5). While random walks and cumulative sums are well established methods for time series changepoint detection in other fields [20, 21, 22], we found no instances where this method was applied in the context of specific growth rate estimation. Furthermore, most prior work uses normally distributed random walks, while we have found Student‐t random walks to yield much clearer results.

In comparison with alternative approaches we found that while analyses with less exact, or even without calibration models may still find the same general trends, a quantitative statement about specific growth rate can only be made with accurate calibrations Figure S1. Observing the popularity of growth rate determination in the bioprocess research community, we view our ${\vec{\mu }}_{t}$ as an important contribution to improve objectivity and reproducibility of this metric. Nevertheless, we would like to remind that with some organisms the biomass/backscatter relationship can depend on morphology, requiring much more sophisticated models, or even making it infeasible to determine growth rate from backscatter at all.

With Section 3.4 we presented a generally applicable method to extract features for machine learning applications from time series data of microbioreactor experiments. By visualizing the high‐dimensional time series features with t‐SNE we showed that the features indeed have the information content needed to reconstruct patterns from the experimental design. The visualization of a high‐dimensional BioLector dataset in a two‐dimensional arrangement that maintains local structure (Figure 8) is just one example of how our bletl package enriches the exploratory data analysis of microbioreactor experiments.

Overall we conclude that Python packages to parse experimental data into standardized data structures are a valuable asset for quantitative, qualitative and exploratory research. As of today, bletl is only able to handle data from BioLector devices. However, it can be extended to other microcultivation devices by implementing additional parser classes. We also welcome contributions and feedback to this open‐source project. For example, more functions for interactive visualizations, tailored to this specific type of datasets, could be added in the future.

## CONFLICT OF INTEREST

The authors have declared no conflict of interest.

## Supporting information

Supporting Information.

## Data Availability

The data is openly available in a public repository (https://github.com/JuBiotech/bletl-paper) and issued a DOI (https://doi.org/10.5281/zenodo.5235460).
