# Supplementary material for: bletl ‐ A Python package for integrating BioLector microcultivation devices in the Design‐Build‐Test‐Learn cycle
Source: Eng Life Sci. 2022 Mar 1;22(3-4):242–59. doi: 10.1002/elsc.202100108 (PMC8961055; doi:10.1002/elsc.202100108)
Supplement: Supplementary file 1 — Supporting Information. [file ELSC-22-242-s001.pdf]

Supplementary Information

4.1 Datasets used in this study

The following dataset files can be found in the `data` directory of the supporting information GitHub repository [25]:

- `8X4PF4.csv` is the raw BioLector dataset.
- `8X4PF4_eventlog.xlsx` contains metadata of induction and sampling events.
- `8X4PF4_medium_composition.xlsx` is the well-wise composition of carbon sources in the growth media.

4.2 Extracted features and t-SNE components

The following files with results from the timeseries feature extraction and embedding are located in the `results` directory of the supporting information GitHub repository [25]:

- `8X4PF4_embedding.xlsx` are the two t-SNE components that were used to arrange data points in the t-SNE visualizations.
- `8X4PF4_extracted_features` contains the raw and cleaned well-wise features extracted from the BioLector dataset.

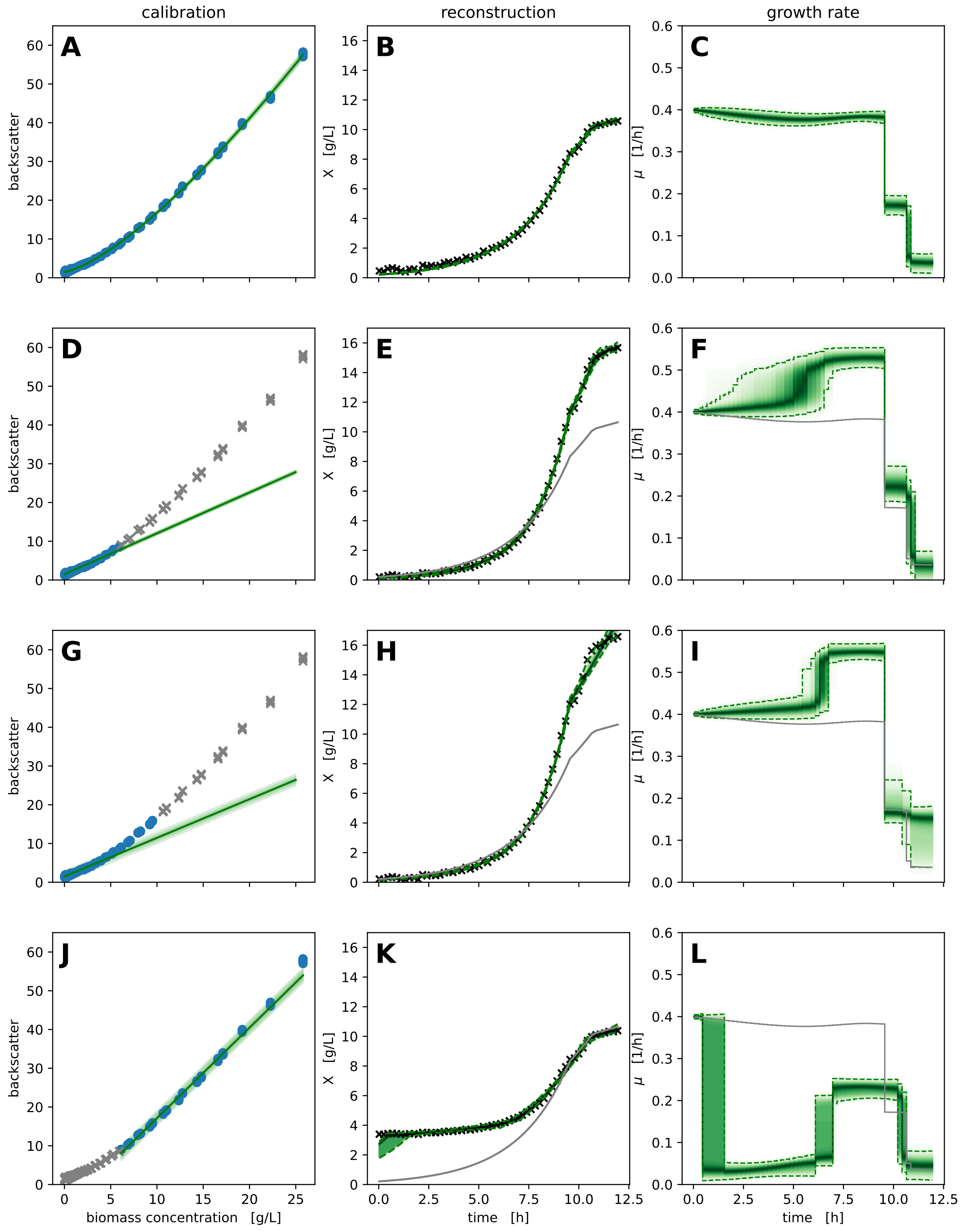

Figure S1: Influence of calibration models on the result of random-walk based $\vec{\mu}_t$ growth rates
An asymmetric logistic calibration model that accurately describes the calibration dataset (**A**) leads to a $\vec{\mu}_t$ profile (**C**) that is used as the baseline (grey) in the other plots. With a linear calibration model fitted only to calibration data up to 6 g/L (**D**) or the expected maximum biomass concentration of 10 g/L (**G**), the growth rate is systematically overestimated (**F, I**). A linear calibration model through only high biomass concentrations (**J**) that would be amenable to individual cell dry weight determination (e.g. >6 g/L leads to an under-estimation of the growth rate, no longer follows the same profile and does not reliably detect switch-points (**K, L**). The green density bands of the calibration models (left column) are likelihood bands showing the expected spread of observations, whereas the density bands in the middle and right column show the posterior probability density, with dashed lines marking the 5 and 95 % percentiles.
